# On the performance of *Sargassum*-derived calcium alginate ion exchange resins for Pb^2+^ adsorption: batch and packed bed applications

**DOI:** 10.1007/s11356-024-33314-w

**Published:** 2024-04-17

**Authors:** Akeem Mohammed, Chantal Mohammed, Andreas Mautner, Matika Kistow, Pooran Chaitram, Alexander Bismarck, Keeran Ward

**Affiliations:** 1 Department of Chemical Engineering, The University of West Indies St. Augustine, St. Augustine, Trinidad and Tobago; 2 Institute of Environmental Biotechnology, IFA-Tulln, University of Natural Resources and Life Sciences Vienna, Konrad-Lorenz-Str. 20, 3430 Tulln, 1180 Vienna, Austria; 3 Institute of Materials Chemistry and Research, Polymer and Composite Engineering (PaCE) Group, Faculty of Chemistry, University of Vienna, Währinger Straße 42, 1090 Vienna, Austria; 4 School of Chemical and Process Engineering (SCAPE), University of Leeds, Leeds, LS2 9JT UK

**Keywords:** Alginate, Bio-based adsorbent, Resource circularity, Adsorption, *Sargassum*

## Abstract

Driven by climate change and human activity, *Sargassum* blooming rates have intensified, producing copious amount of the invasive, pelagic seaweed across the Caribbean and Latin America. Battery recycling and lead-smelter wastes have heavily polluted the environment and resulted in acute lead poisoning in children through widespread heavy metal contamination particular in East Trinidad. Our study details a comprehensive investigation into the use of *Sargassum* (*S. natans*), as a potential resource-circular feedstock for the synthesis of calcium alginate beads utilized in heavy metal adsorption, both in batch and column experiments. Here, ionic cross-linking of extracted sodium alginate with calcium chloride was utilized to create functional ion-exchange beads. Given the low quality of alginates extracted from *Sargassum* which produce poor morphological beads, composite beads in conjunction with graphene oxide and acrylamide were used to improve fabrication. Stand-alone calcium alginate beads exhibited superior Pb^2+^ adsorption, with a capacity of 213 mg g^−1^ at 20 °C and pH 3.5, surpassing composite and commercial resins. Additives like acrylamide and graphene oxide in composite alginate resins led to a 21–40% decrease in Pb^2+^ adsorption due to reduced active sites. Column operations confirmed Alginate systems’ practicality, with 20–24% longer operating times, 15 times lower adsorbent mass on scale-up and 206% smaller column diameters compared to commercial counterparts. Ultimately, this study advocates for *Sargassum*-based Alginate ion-exchange beads as a bio-based alternative in Trinidad and developing nations for dealing with heavy metal ion waste, offering superior heavy metal adsorption performance and supporting resource circularity.

**Graphical Abstract:**

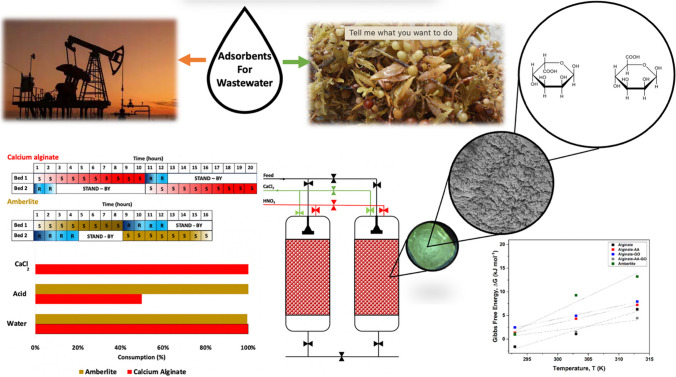

**Supplementary Information:**

The online version contains supplementary material available at 10.1007/s11356-024-33314-w.

## Introduction

Heavy metal pollution has significantly impacted both the environment and human health due to modern industrial growth and resource depletion, particularly affecting developing countries. Arsenic (Ar), cadmium (Cd), chromium (Cr), copper (Cu), lead (Pb), nickel (Ni) and zinc (Zn) are commonly found in wastewater, originating from various natural and anthropogenic activities like mining and fossil fuel burning (Lambert et al. 2000; Gao et al. 2020). Lead contamination in East Trinidad stems from ongoing exposure to lead from battery recycling and lead-smelter wastes, resulting in acute poisoning in children and significant environmental damage, particularly in sensitive areas like rivers and wetlands, necessitating immediate remedial measures to mitigate risks to public health and prevent further contamination (LoopTT 2018).

These metals accumulate in soil, water and organisms, leading to bioaccumulation and long-term contamination, posing risks to humans, plants and animals by interacting with cellular DNA (Tchounwou et al. 2012). The consequences extend to ecosystems, agriculture and human health, causing disruptions in nutrient cycling, biodiversity loss, decreased productivity and health issues such as neurological damage and increased cancer risk (Alengebawy et al. 2021; Balali-Mood et al. 2021). Various techniques such as chemical precipitation, ion exchange and adsorption can mitigate heavy metal contamination in wastewater, but conventional methods have limitations including inefficiency at low concentrations, lack of selectivity, pH sensitivity, generation of secondary pollutants, long processing times and high costs (Fu and Wang 2011; Rajasulochana and Preethy 2016). These challenges underscore the need for innovative and cost-effective solutions to address heavy metal pollution and safeguard environmental and human health on a global scale.

Adsorption is one technique that is widely used due to its simplicity, high removal efficiency and adaptability in design. The most effective adsorbents employed in wastewater treatment for heavy metal removal to date have been activated carbon and synthetic polymer resins (Qin et al. 2020). However, due to the high cost and energy-intensive regeneration of activated carbon, its use is less economically appealing on an industrial scale. Furthermore, these adsorbents, despite having excellent adsorption capacities for certain heavy metal ions, have disadvantages, such as undesirable non-biodegradability and causing secondary contamination (Si et al. 2022). Therefore, for a transition to more sustainable wastewater treatment, it is fundamentally important to innovate new, greener methods of heavy metal ion adsorption and easy regeneration through the use of bio-based adsorbents.

Composites based on chitosan and cellulose derivatives, including cellulose filament fibers, cellulose nanofibrils, nanocellulose, cellulose nanocrystals and microcrystalline cellulose, are prevalent bio-based adsorbents utilized for heavy metal remediation (Boccia et al. 2024). Cellulose, characterized by its high specific surface area, surface functionalization, aspect ratio, mechanical strength, chemical inertness and widespread availability, along with hydroxyl and epoxy groups, enables effective heavy metal ion adsorption primarily through mechanisms such as ion exchange (Pandey et al. 2023). Conversely, chitosan exhibits remarkable chelating properties, pH responsiveness and contains amine and hydroxyl groups, facilitating robust coordination bonds with heavy metal ions (Verma and Quraishi 2021).

Similarly, calcium alginate derived from brown seaweed can act as a bio-based adsorbent for efficient heavy metal adsorption due to surface interactions facilitated by abundant hydroxyl and carboxyl groups. Its renewability, biocompatibility and ease of regeneration make it promising for metal ion removal (Gao et al. 2020; Adewuyi 2020; Benettayeb et al. 2022). However, to enhance its effectiveness, composite technology incorporating additives like acrylamide (AA) and graphene oxide (GO) is utilized to improve morphological characteristics and mechanical stability, thus increasing ion-exchange efficiency (Craciun et al. 2019; Jiao et al. 2016; Tang et al. 2020). These additives create deeper pores and smoother surfaces, enhancing ion permeability and adsorption rates (Algothmi et al. 2013; Zhao et al. 2016). Despite the potential, the bio-based adsorbent industry is at a low Technology Readiness Level (TRL), necessitating further development to ensure commercial viability (Udugama et al. 2017). Thus, there are ongoing efforts focusing on refining alginate structures to address inherent limitations and maximize adsorption capacity, aligning with the imperative for sustainable and effective heavy metal remediation methods (Li et al. 2023; Zeng et al. 2023; Dong et al. 2023; Abd-Elhamid et al. 2022). However, it should be noted that the alginate utilized in these studies is from pure commercial sources of alginate but there is limited research on the utilization of *Sargassum natans* as a bio-based adsorbent for heavy metal ion remediation in a developing nation such as Trinidad.

Within the past 12 years, the Caribbean and Latin America have seen massive onslaughts of *Sargassum* natans (brown seaweed) onto its shores, negatively impacting marine ecosystems resulting in a decrease in economic activities (Milledge and Harvey 2016). There is a great need for more environmentally conscious waste management utilization strategies for the *Sargassum* as disposal techniques involve incineration and decomposition adding to further greenhouse gas (GHG) emissions and air pollution within the Caribbean and Latin America. However, this waste seaweed has been shown to be an effective supply chain for sodium alginate production (Mohammed et al. 2018; Mohammed et al. 2020a, b) and as such, offers a plethora of uses (López-Miranda et al. 2023; Mohammed et al. 2023a, 2023b, 2019). However, in comparison to alginates produced from commercial seaweed sources such as *Laminaria*, *Sargassum*-derived alginate is of a low yield and quality in terms of purity (Mohammed et al. 2020a) and as such is better suited for use in remediation of heavy metals as opposed to food and pharmaceutical value chains. Our past work has illustrated the appropriateness and applicability of calcium alginate thin films from *Sargassum* natans as a successful biosorbent for the adsorption of heavy metal ions with a metal selectivity of Pb > Cu > Cd (Mohammed et al. 2019, 2022).

Thus, the novelty of this study lies in utilizing *Sargassum* as a waste management strategy to fabricate calcium alginate bio-sorbents to act as a remediation tool for dealing with heavy metal ion waste in rural communities affected in East Trinidad and developing nations. Furthermore, the advantage of this study is that it extends the utilization of the pelagic biomass as a resource circular supply chain for the creation of useful products and presents a new *Sargassum*-based adsorbent within the area of wastewater treatment and further development of the bio-based adsorbent industry. Thus, here batch and column experiments were employed to (i) investigate the performance of *Sargassum*-derived bio-sorbents for heavy metal ion adsorption using Pb^2+^ as a case study and (ii) to explore the impacts of additives on the efficiency of the adsorption process. Batch-wise experiments served to understand the kinetics behind adsorption and column experiments were performed in order to evaluate industrial potential and applicability. Ultimately, our results are compared against the performance of commercial synthetic adsorbents to identify the potential trade-offs, drawbacks and solutions to its wider commercial adoption.

## Experimental

### Materials and equipment

All reagents used were of analytical grade and all solutions were prepared using deionized water. The calcium alginate beads were fabricated using sodium alginate extracted from *S. natans*. The seaweed pre-treatment process was done using formaldehyde (BDH, 36.5 wt. % in water). Acid treatment was carried out using sulphuric acid (J.T. Baker, 96.4%). For the alkaline extraction process, sodium carbonate (Scharlau, 99.9%) was used. Bleaching was done using sodium hypochlorite (Alfa Aesar, 11–15% available chlorine). For the purification process of the alginate, 50% (w/v) alcohol (BDH, 94–96% ethanol and methanol) was used. Calcium chloride dihydrate (ACS, USA > 95% purity) was used as the cross-linker in fabricating the beads. A peristaltic pump (Leadfluid, BT100F), equipped with a rubber tube and needle (*d* = 0.34 mm), was used for resin extrusion. A freeze drier (Armfield SB4) was used for drying calcium alginate adsorbents. Methylene bisacrylamide (AA, Sigma Aldrich, > 99% purity) and ammonia functionalized graphene oxide (GO, Sigma Aldrich, 1000 ppm) were used as additives to enhance the performance of the primary extracted alginate biopolymer. Amberlite IR-120 (Sigma Aldrich) was used as a commercial adsorbent standard for performance comparison. A 1 M Pb^2+^ stock solution was used for preparing analytical standards (Analytik Jena, USA). Pb(NO_3_)_2_ (Sigma Aldrich, > 99% purity) was digested and used to simulate waste water contaminated with Pb^2+^ ions. The equilibrium Pb^2+^ concentration was determined using atomic absorption spectroscopy (AAS, Analytik Jena GmbH novAA 300, Germany). NaOH (VWR Life Science, > 99% purity) pellets were used for pH adjustment. A vortex mixer (WVR) and incubator (WVR) were used to maintain experimental homogeneity. Sixty-five to seventy percent HNO_3_ (JT Baker) was used for adsorbent bed regeneration process and adjusting pH. Scanning electron microscopy (SEM) was carried out using Denton Deskll sputter coater and JEOL JCM-6000 electron microscope (Neoscope, Freising, Germany). Prior to imaging, the samples were sputter-coated with a layer of gold (Jeol JFC-1200 Fine Coater, Freising, Germany). X-ray photoelectron spectroscopy (XPS) was performed with a Nexsa XPS system (Thermo-Fisher). Inverse gas chromatography (IGC, Surface Energy Analyzer, Surface Measurements Systems Ltd., U.K.) was used to determine the specific surface area of the beads. FTIR spectra were obtained using a Perkin Elmer Spectrum 400 FT-IR/FT-NIR spectrometer (USA) with a universal ATR sampling accessory.

### Alginate extraction from *S. natans*

Sodium alginate was extracted from *S. natans* (yield = 28%, M/G ratio = 0.45, molecular weight = 3.14–3.2 × 10^5^ g mol^−1^) as previously reported by us (Mohammed et al. 2018, 2020a)*.* Briefly, *S. natans* was continuously washed to remove debris, salt and sand. After the washing step, the *Sargassum* was preserved in 2% (w/v) formaldehyde solution overnight to remove all phenolic compounds. The seaweed was then rinsed, dried and pulverized (505 $\mathrm{\mu m})$. The pulverized seaweed was then reacted with 0.5 M H_2_SO_4_ (1:15 v/v) at 40 °C for 1 h. After 1 h, the reactant mixture was centrifuged at 8000 rpm for 10 min. The precipitate was then reacted with 3.75 wt.% Na_2_CO_3_ solution (1:12.3 v/v) for 6 h at 80 °C. The mixture was centrifuged and the solid fraction was then contacted with fresh alkali solution in a second-stage extraction using the same process conditions as the first. The supernatant (crude alginate) was then bleached using a 1:50 (v/v) excess volume of NaOCl to crude alginate. After this step, 1 M H_2_SO_4_ was added until the pH of the solution dropped to 2.0. This solution was centrifuged and the supernatant gel was collected. This gel was dissolved in 50 wt.% ethanol for 2 h. Five weight percentage Na_2_CO_3_ was subsequently added until the pH increased to 10.0. The mixture was centrifuged and the alginate gel collected and freeze-dried at − 41 °C for 24 h, giving pure sodium alginate powder (purity $\ge$ 95%). The purity control was done using high-performance liquid chromatography (HPLC) with a C18 stationary phase column (150 × 4.6 mm i.d., 3.5 μm) and buffer solution mobile phase.

### Adsorbent fabrication

To obtain better bead formation and to evaluate the performance of composite beads compared to pure calcium alginate materials, scoping of additives presented in literature was carried out and resulted in the selection of acrylamide (AA) and ammonia functionalized-graphene oxide (GO) (Craciun et al. 2019; Jiao et al. 2016; Tang et al. 2020). This study comprised of 4 types of beads: pure calcium alginate beads (Alginate) and composite beads calcium alginate/acrylamide (Alginate-AA), calcium alginate/graphene oxide (Alginate-GO) and calcium alginate/acrylamide/graphene oxide (Alginate-AA-GO). Preliminary rheology experiments were conducted to determine the amounts of each additive required to achieve bead sphericity > 70% (Supplementary information). Pure calcium alginate beads were produced by dissolving extracted sodium alginate from *S. natans* (4.25% w/v) in deionized water. One hundred milliliters of this solution was extruded using a peristaltic pump equipped with a rubber tube and needle (*d* = 0.34 mm) and added dropwise into 8.5% (w/v) CaCl_2_ solution agitated at 200 rpm. The mixture was left to cross-link for 2 h. The newly fabricated beads were then rinsed with deionized water for a further 2 h, air-dried overnight for 48 h and then freeze-dried (Armfield SB4) at – 45 °C. Similarly, all composite beads were fabricated by adding the various additives to the alginate solutions as follows: Alginate-AA were prepared by adding 2.125% (w/v) AA, Alginate-GO was prepared by adding 15% (v/v) GO and Alginate-AA-GO was made by adding 2.125% (w/v) AA and 15% (v/v) GO.

### Batch adsorption experiments

The performance of the calcium alginate absorbents was evaluated against a commercial alternative, Amberlite IR-120 (Amberlite). 100, 250, 500, 750 and 1000 ppm Pb^2+^ stock solutions were prepared via acid digestion of Pb(NO_3_)_2_ with 1% (v/v) HNO_3,_ adjusted to pH 3 using NaOH pellets (Mohammed et al. 2022). Ten milliliters of 100 ppm Pb^2+^ solution and 0.05 g of alginate beads (loading = 5 g L^−1^) (Tsekova et al. 2010) were added to a conical flask and vortexed (VWR) for homogeneity. Samples were then incubated at 20 °C for 24 h and agitated at 100 rpm. Afterwards, a 5-mL sample was diluted to fit the analytical testing range (0.1–5 ppm) and analysed using atomic absorption spectroscopy (AAS). The overall procedure was repeated for all concentrations at 30 °C and 40 °C. Finally, batch experiments were repeated in triplicate for each adsorbent material (Alginate-AA, Alginate-GO, Alginate-AA-GO and Amberlite). All results were taken within a coefficient of variance of < 5%.

### Adsorption isotherms and thermodynamic performance

A mass balance approach was utilized to calculate the total mass of Pb adsorbed per mass of adsorbent shown in Eq. (1).

1 $${q}_{e}=({C}_{o}-C)\times \frac{V}{RMM\times m}$$

${q}_{e}$ (mg/g) is the mass of metal ions adsorbed per unit mass of adsorbent and ${C}_{o}$ (mg/L) and $C$ (mg/L) denote the initial and final concentrations of the solution, respectively. m (g) is the mass of adsorbent and $V$ (L) denotes the volume of the heavy metal ion solutions.

In characterizing the adsorption phenomena of all adsorbents, both Langmuir and Freundlich isotherms were used; however, only details of the Langmuir model are presented here as it was found to be the most appropriate for the adsorbents investigated. Details of the Freundlich model are presented in the Supplementary Data file. The Langmuir isotherm was subsequently used to model the adsorption performance using Eqs. (2)–(5). Langmuir isotherms have been shown to accurately model the adsorption of heavy metal ions onto calcium alginate surfaces (Fadl 2023; Khamseh et al. 2023; Papageorgiou et al. 2009). The equilibrium constant K_L_ was determined Eq. (2). This was subsequently used to determine the Gibbs Free Energy, enthalpy and entropy associated with the ion-exchange phenomena.

2 $${q}_{e}= \frac{{q}_{max}{K}_{L}{C}_{e}}{1+ {K}_{L}{C}_{e}}$$

3 $$\frac{1}{{q}_{e}}=\frac{1}{{q}_{max}{K}_{L}}\left(\frac{1}{{C}_{e}}\right)+ \frac{1}{{q}_{max}}$$

4 $$\Delta G=-RTln{K}_{L}$$

5 $$\Delta {\text{G}}=\Delta {\text{H}}-{\text{T}}\Delta {\text{H}}$$

${C}_{e}$ is the equilibrium concentration remaining in solution (mg L^−1^ of aqueous solution), ${q}_{max}$ is the maximum monolayer capacity (mg g^−1^), ${K}_{L}$ is the equilibrium constant (Don and Green 2008), ΔH (kJ mol^−1^) is the binding enthalpy, ΔS (J mol^−1^ K^−1^) is the entropy, R (kJ mol^−1^ K^−1^) is the universal gas constant and ΔG (kJ mol^−1^) is the Gibbs Free Energy at the reaction temperature T (K).

### Packed bed application and scale-up

**Fig. 1:**
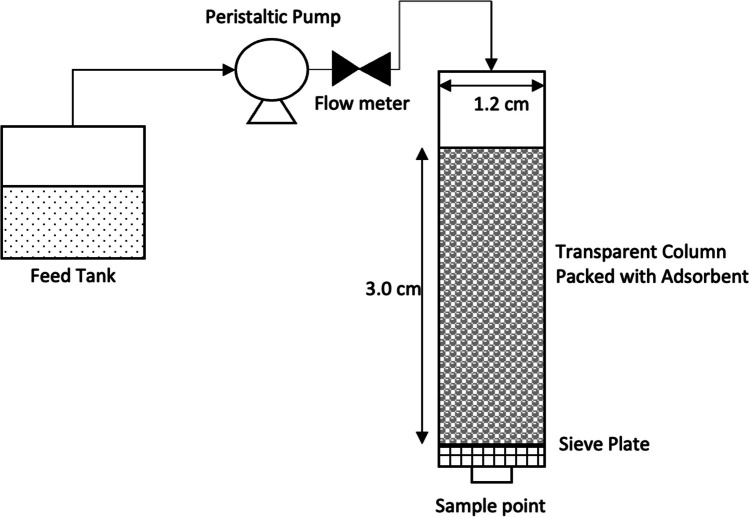
Schematic for the packed bed set-up used to investigate the adsorbent performance

To investigate the industrial applicability of alginate materials for Pb adsorption, the best-performing adsorbent arising from batch experiments was compared to Amberlite using a packed bed ion-exchange system. Each adsorbent was packed separately into transparent cylindrical columns with a diameter of 1.2 cm and bed height of 3 cm in accordance with Sami et al. (2022). A peristaltic pump with a flow meter was used to pump fresh feed into the top of the column as shown in Fig. 1. The column was fed in a downward flow direction to withdraw any trapped air between the beads and control the rate of flooding in the column (Sami et al. 2022).

Initial trials with varying Pb^2+^ concentration were conducted until a sigmoidal breakthrough curve was obtained. To prevent channelling and flooding within the column, operating conditions were pre-determined using deionized water for each adsorbent material. Approximately 150 ppm (C_I_) of Pb^2+^ solution was pumped into the column at a flow rate of 8 mL min^−1^. Samples were taken within 1-min to 1-h time intervals from the base of the column, diluted and analysed using AAS (C_F_) until each column was exhausted (C_F_ > 90% C_I_). Performance curves were plotted to determine breakthrough time and the exhaustion period.

As part of this preliminary assessment on the comparison of adsorption efficiency of *Sargassum*-based alginate systems to commercial adsorbents, the Yoon-Nelson model (Eq. (5)) was used to fit the experimental data as it determines the breakthrough parameters (Naushad et al. 2019), and does not require any information regarding the adsorbate, the type of adsorbent or physical characteristics of the bed (Chittoo and Sutherland 2020), which aligns with our experimental methodology. As these alginate systems are refined and better understood in terms of performance, a more detailed analysis of their adsorption characteristics considering advanced breakthrough models and characteristic mass transfer limitations will be explored as part of our future research. Equation (6) and Eq. (7) were used to determine the specific intrinsic kinetics of the packed bed adsorption for each adsorbent material (Giannakas 2017).

6 $$\frac{{C}_{F}}{{C}_{I}}= \frac{1}{1+expexp [k( \tau -t)}$$

7 $$ln\frac{{C}_{F}}{{C}_{I}-{C}_{F}}=kt-\tau k$$

8 $${q}_{e}=\frac{{C}_{I}Q \tau }{m}$$

The linear form of the Yoon-Nelson is given in Eq. (7) where k is the Yoon–Nelson proportionality constant (min^−1^) and τ is the time required for retaining 50% of the initial sorbate (min) also referred to as breakthrough. The values of k and τ can be determined from the slope and intercept of a plot of ln(C_F_/C_I_ − C_F_) versus t. Equation (8) was used to calculate the amount of Pb^2+^ removed per unit mass of adsorbent q_e_ (mmol g^−1^) of a mass m (g) at a flowrate Q (L h^−1^), and initial concentration C_I_ (mmol L^−1^). Furthermore, the Pb^2+^ removal efficiency was determined using the breakthrough (REM_b_, %), exhaustion (REM_e_, %) points and the mass transfer zone length (H_MTZ_, cm), according to Eqs. (9–13) respectively (da Costa et al. 2022).

9 $${q}_{B}= \frac{{C}_{I}Q}{W}{\int }_{0}^{{t}_{B}}(1-\frac{{C}_{F}}{{C}_{I}}) dt$$

10 $${q}_{E}= \frac{{C}_{I}Q}{W}{\int }_{0}^{{t}_{E}}(1-\frac{{C}_{F}}{{C}_{I}}) dt$$

11 $${REM}_{b}=\left(\frac{{q}_{B} W}{{C}_{o} Q {t}_{B}}\right)100$$

12 $${REM}_{e}=\left(\frac{{q}_{E} W}{{C}_{o} Q {t}_{E}}\right)100$$

13 $${H}_{MTZ}=\left(1- \frac{{q}_{B} }{{q}_{E}}\right){H}_{L}$$

where q_B_ is the amount of Pb adsorbed onto each adsorbent at t_B_ (mg g^−1^), q_E_ is the amount of Pb adsorbed onto each adsorbent at t_E_ (mg g^−1^), W is the mass of beads (g), Q is the Pb^2+^ solution flow rate (L/h), t_B_ (h) is the recorded time when C_F_ = 0.05, H_L_ is the bed height (cm) and t_E_ (h) is the recorded time when C_F_ = 0.95.

The column experiment was carried out in triplicates. Bed regeneration was performed during cycles by pumping 1% (v/v) HNO_3_ at 8 mL min^−1^ through the bed until a concentration (C_F_) < 3 ppm was observed at the column exit. Ultimately, the parameters determined from these models were used to scale-up the process and make deductions about the performance on an industrial scale.

### Characterization of adsorbents

#### Inverse gas chromatography (iCG)

Inverse gas chromatography (Surface Energy Analyzer, Surface Measurements Systems Ltd., U.K.) was used to analyse the specific surface area (SSA) at 30 °C and 0% RH. Samples of about 300 mg were packed into glass columns (inner Ø 4 mm, outer Ø 6 mm). The columns were plugged with glass wool. Octane was used as probe and flown (10 sccm) over the samples, and the retention times and coverages were recorded using a flame ionization detector (FID). From these values, the SSA was computed for a range of relative pressures (between 0.05 and 0.3) from the centre of mass of the peaks using the BET model.

#### Scanning electron microscopy (SEM)

The morphologies of the samples were studied using SEM (JEOL JCM-6000). Prior to imaging, the samples were sputter coated (Jeol JFC-1200 Fine Coater) with a layer of gold for 30 s at a coating current of 30 mA.

#### X-ray photoelectron spectroscopy (XPS)

XPS (Nexsa, ThermoFisher) was performed using an integrated flood gun, radiation source gun-type Al Kα operating at 72 W and a pass energy of 200 eV, a spot size of 400 μm, “Standard Lens Mode”, CAE Analyser Mode and an energy step size of 1 eV for the survey spectra (40 passes). Prior to analysis, the surface was cleaned with Ar-clusters (1000 atoms, 6000 eV, 60 s). The high-resolution spectra (step size 0.1 eV) of the single elements were acquired with 40 passes at pass energies of 50 eV.

#### Fourier transform infrared (FTIR) spectroscopy

FTIR spectra were collected in the range of 650 to 4000 cm^−1^ with a resolution of 4 cm^−1^. A total of 64 scans were used to obtain each spectrum. The IR-laser wavenumber was set at 15780 cm^−1^, OPD velocity of 0.20 cm s^−1^ and J-stop size of 8.94 mm.

## Results and discussion

### Characterization of the adsorbents

#### Morphology and specific surface area by SEM and iCG

**Fig. 2:**
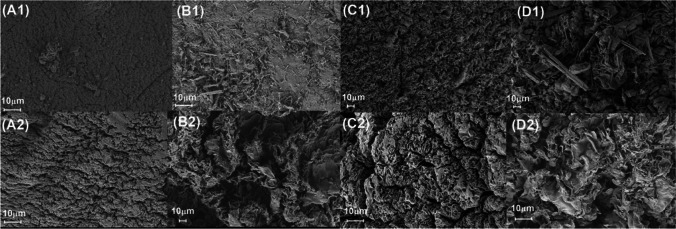
Morphology of various calcium alginate bead blends. (1) Virgin and (2) post-adsorption surfaces for **A** Alginate, **B** Alginate-AA, **C** Alginate-GO and **D** Alginate-AA-GO

Blending of alginate with additives can result in interactions which alter, at best improve, surface properties such as the specific surface area (SSA). SEM was used in conjunction with iGC to observe these changes and quantify the impacts on the SSA. Figure 2 shows the adsorbent surfaces before (A) and after (B) adsorption. Pure alginate’s surface appears uniform with microporous ridges (Fig. 2(A1)); however, when single additives are incorporated into the alginate matrix (Fig. 2(B1) and (C1)), agglomeration is observed. For AA, non-uniform macro-structures are distributed chaotically resulting in a low SSA of 0.374 m^2^ g^−1^ (Table 1) (Tripathy and Singh 2001). For GO, deeper more defined pores were observed (Fig. 2(C1)) facilitating the highest SSA of 0.412 m^2^ g^−1^ (Algothmi et al. 2013). Composite beads containing both AA and GO (Fig. 2(D1)) exhibit a surface which appears most chaotic and non-uniform, mediated by the interaction of acryl amide and graphene oxide also yielding the lowest SSA (0.300 m^2^ g^−1^).

**Table 1:** Specific surface area and composition for the various blends of adsorbents

Adsorbent	Surface area (m^2^ g^−1^)
Alginate	0.397
Alginate-AA	0.374
Alginate-GO	0.412
Alginate-AA-GO	0.300

After adsorption, minimal changes were observed in the morphological characteristics of all bead types. Accompanying adsorption, a deepening of these ridges was observed resulting in an increase in homogeneity (Fig. 2A–D).

#### Chemical composition and functionality by XPS and FTIR spectroscopies

**Fig. 3:**
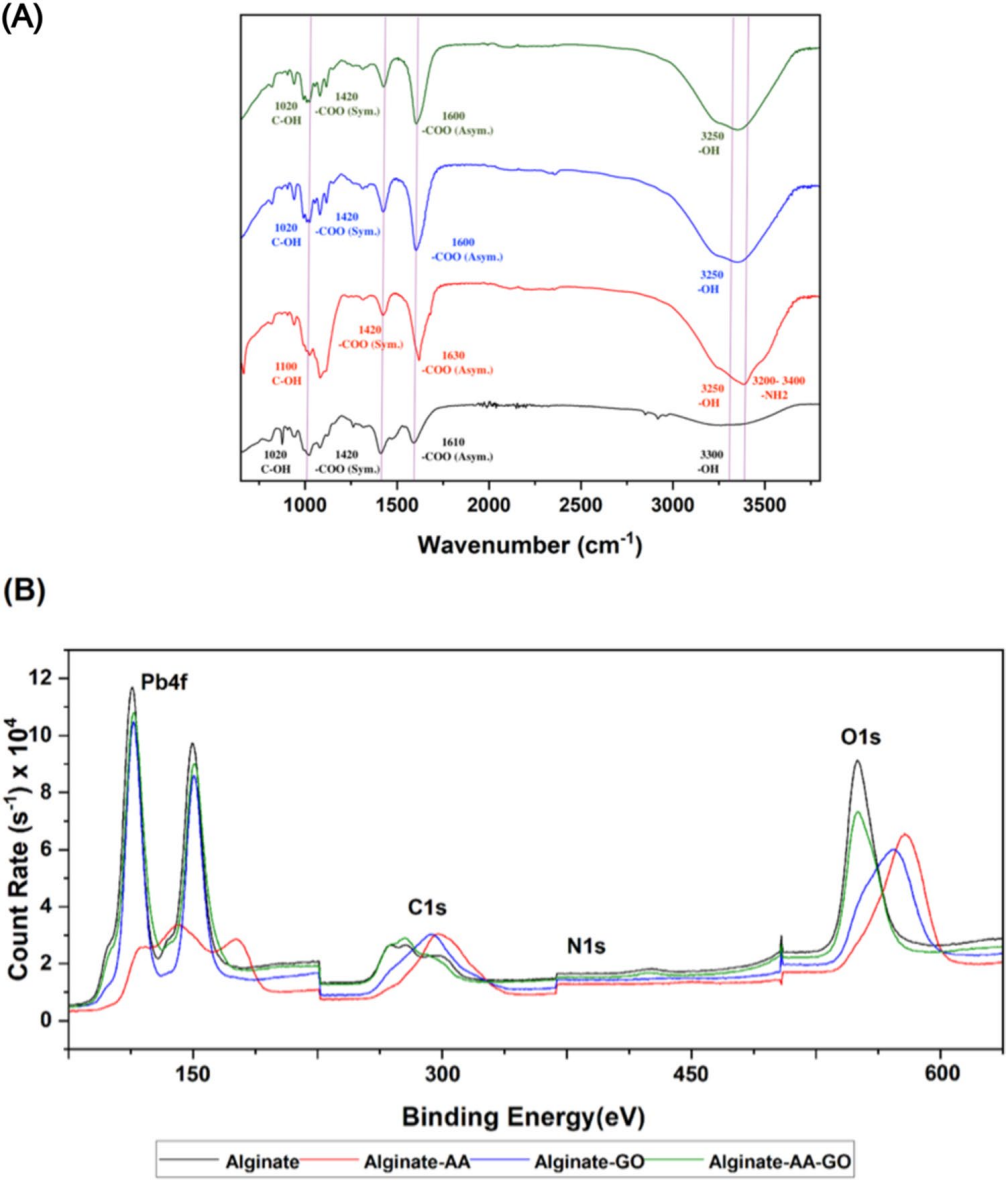
**A** FTIR and **B** XPS survey spectra for post-adsorption calcium alginate adsorbents with additives

The binding of metal ions to adsorbents is driven by the availability of binding sites. FTIR spectroscopy was carried out on the Alginate system and composite beads in order to evaluate changes in functionality. The asymmetrical stretching vibration of COO^−^ at 1610 cm^−1^ in calcium alginate shifts to the right (1630 cm^−1^) for the system containing AA (Fig. 3A). This suggests the formation of new hydrogen bonds between the COO^−^ groups of sodium alginate and CONH_2_ groups of acryl amide. Additionally, at 3400 cm^−1^ and 3200 cm^−1^, the stretching vibration of NH_2_ groups involved in both inter and intramolecular hydrogen bonds sharpened and shifts to the right. This implies the presence of hydrogen bonds between the OH groups of sodium alginate and the NH_2_ groups of acrylamide (Şolpan and Torun 2005).

The interaction between calcium alginate and graphene oxide results in an increase in intermolecular hydrogen bonding and electrostatic interactions connected to the addition of ammonia functionality. This was shown by the shift of the OH and COO^−^ peaks to 3250 cm^−1^ and 1600 cm^−1^, respectively (Nie et al. 2015). Furthermore, for the system containing both GO and AA, the peak at 3250 cm^−1^ drastically diminishes and broadens indicating the loss of OH groups and intercalated water molecules as a result of the copolymerization of AA and GO. However, the Alginate-GO-AA system appears to be dominated by the interactions between alginate and GO with minor shifts due to the interaction of GO and AA.

For the alginate system, the C1s peak was detected at 284 eV shifting to the right to 288 eV with the addition of AA which was attributed to the interactions between C and N, i.e., between acrylamide and alginate. This peak shift was explained by the presence of O = C – N functionalities (Liu et al. 2012). As acrylamide coexists with sodium alginate solution during cross-linking, macroscopic entanglement of the polymeric networks occurs during gelation. Hence, C = C is converted to C–C. This C of the acrylamide chain interacts with the oxygen ion of the -COO-, resulting in a decrease in the free carboxyl group (Pragya et al. 2021). Furthermore, a small N1s peak appears at 400 eV for the AA composite mainly due to the presence of amide bonds -NH_2_.

The N peak is also present for the GO composite due to the ammonia functionality NH_3_ (E et al. 2020). However, N1s peaks are absent for the pure Alginate system and AA-GO alginate as expected as there are no N containing binding sites present on the surface of these beads. As AA and GO interact, nitrogen from the AA monomer results in N-doping yielding the formation of heterocyclic nitrogen. This formation changes the structure of GO; hence, the heterogeneity of the composite may have resulted in N being not detected in the elemental surface composition (Jin et al. 2019). The O peak at approximately 555 eV for the alginate and AA-GO composite bead exhibited similar binding energies, while for AA and GO composites the peak was shifted to 585 and 590 eV, respectively.

### Adsorption behaviour of alginate beads

**Fig. 4:**
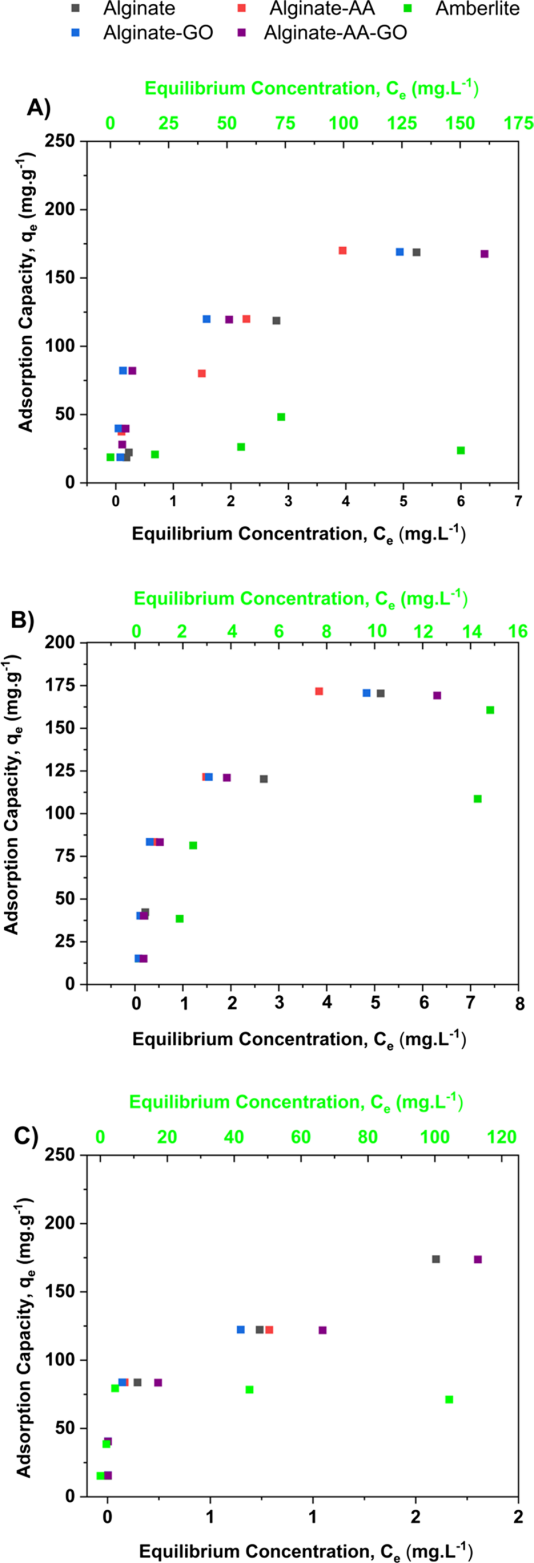
Adsorption isotherms for each alginate-based adsorbents examined at **A** 20 °C, **B** 30 °C and **C** 40 °C. black square: Alginate, red square: Alginate-AA; blue square: Alginate-GO; gray square: Alginate-AA-GO; green square: Amberlite

The effect of the thermodynamics on the adsorption capacity (q_e_) of various alginate composites was studied using batch experiments, carried out at various temperatures. The adsorption characteristics are shown in Fig. 4. For all plots, q_e_ increases as the equilibrium concentration; C_e_ increases until a saturation point is reached. At this point, all available adsorption sites are filled and no longer any more adsorbate can be accommodated. This is characteristic of a Langmuir-type adsorption process (model results given in Table 2) and suggest that monolayer sorption occurs on the surface of the alginate beads (Ajaelu et al. 2021). The alginate system outperforms the other beads, indicating more readily available active sites for Pb^2+^ adsorption (Fig. 4).

**Table 2:** Mean parameter data for adsorption isotherms for Pb^2+^ onto Alginate, alginate composites and commercial resin

Model parameter	Maximum adsorption capacity, q_max_ (mg g^−1^)			Langmuir constant, K_L_			R^2^		
Temperature (°C)	20	30	40	20	30	40	20	30	40
Alginate	213	170	101	330	11.2	1.55	0.99	0.99	0.96
Alginate-AA	193	124	105	81.0	16.2	1.73	0.94	0.99	0.99
Alginate-GO	172	156	119	280	21.0	2.76	0.98	0.99	0.99
Alginate-AA-GO	179	164	116	286	5.54	1.60	0.99	0.99	0.92
Amberlite	78.1	61.4	25.0	40.0	1.63	1.52	0.94	0.95	0.94

The Alginate system exhibited the highest adsorption capacity of 213 mg g^−1^ at a temperature of 20 °C. This was attributed to the higher availability of COO^−^ protonation sites, which increases the electronegativity of the alginate dimer junction and negative surface charge, thus supporting ion-exchange (Ibáñez and Umetsu 2002). It was also noted that incorporation of additives resulted in a reduction of the adsorption capacity by 21%, 34% and 40% for AA, GO and AA-GO systems, respectively. This reduction in binding of Pb^2+^ ions linked to the interactions between additives and the alginate surface, compromising ion-exchange propensity as confirmed through characterization previously detailed in the “Characterization of the adsorbents” section.

The uptake of divalent ions by alginate systems occurs in stages. The first is the interaction of Pb^2+^ ions with COO^−^ residues on a single guluronate, forming monocomplexes as a result of chelation. This is followed by the propagation of egg-box dimers (second stage) due to specific pairing of two parallel chains, resulting in the incorporation of Pb^2+^ ions within the alginate matrix (Mohammed et al. 2019). As additives are added to the alginate system, inhibition takes places between Pb^2+^ ions for the active sites occupied with these additives. This reduces cooperative binding resulting in a decrease in total metal ion uptake. Amongst all the additives tested, adsorption performance follows AA > GO > AA-GO.

For Amberlite IR-120, the main functional group associated with Pb adsorption was ${SO}_{3}^{-}$ within a styrene DVB matrix. The material is a microporous, strong acidic cationic resin with an operating pH of 0 – 14 and a maximum operation temperature of 150 °C (Meshram et al. 2013). The maximum adsorption capacity of Pb^2+^ reported for Amberlite is 62.4 mg g^−1^, similar to the maximum adsorption capacity of 61.4 mg g^−1^ obtained in our work (Table 2) at 30 °C for a contact time of 24 h (Tabatabaei et al. 2013).

### Thermodynamic properties of alginate beads

**Fig. 5:**
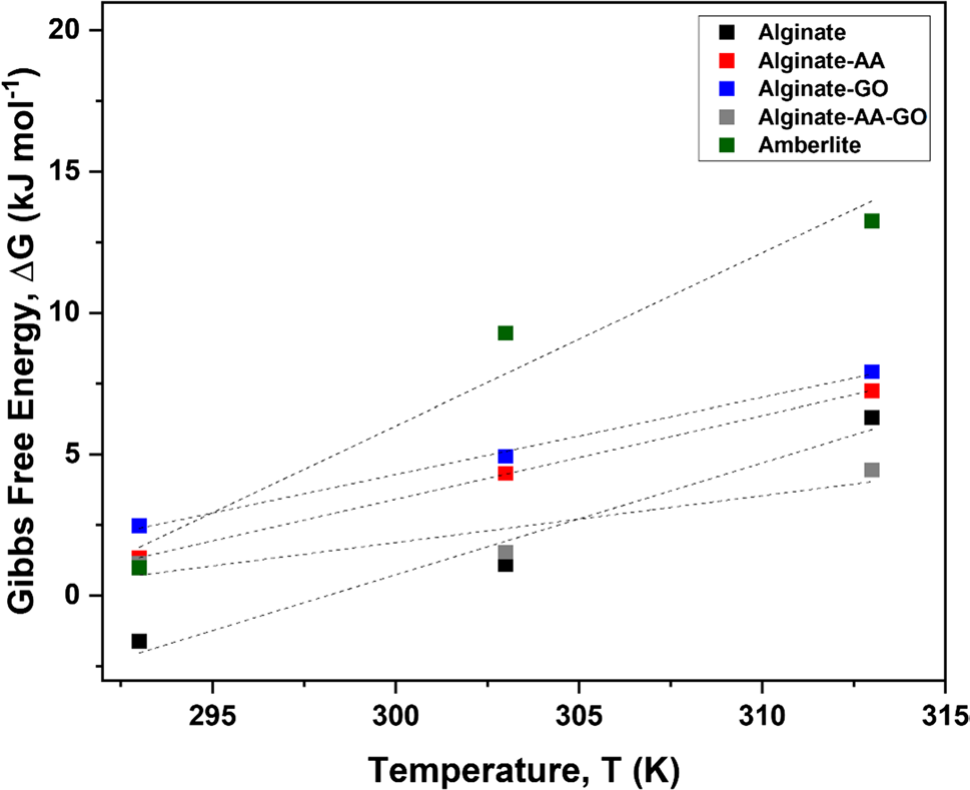
Gibbs free energy (kJ/mol), temperature (K) relationship for calcium alginate beads, composite beads and commercial resin. Black square: Alginate; red square: Alginate-AA; blue square: Alginate-GO, gray square: Alginate-AA-GO, green square: Amberlite

The adsorption of Pb^2+^ onto the binding sites of various adsorbents involve the formation of bonds, which results in the absorbance or emittance of energy from/to the bulk environment. Thus, the thermal environment of a reaction can play a pivotal role in the reaction mechanism itself (Braia et al. 2017). Thermodynamic studies can be used to understand the energy transformations taking place during a reaction and can provide deductions as to the optimal environment needed for the reaction as well as the extent of the reaction. It was observed that the Gibbs free energy notably increases with increasing temperature for all resins (Fig. 5, Table 2), explaining the highest maximum adsorption capacity for all adsorbents at 20 °C (Alginate = 213 mg g^−1^, Alginate-AA = 192 mg g^−1^, Alginate-GO = 172 mg g^−1^, Alginate-AA-GO = 179 mg g^−1^). Additionally, it was observed that K_L_ is reduced at higher temperature, which illustrates lower interactions between adsorbate and the surface (Torres-Caban et al. 2019), resulting in poor metal uptake. This correlates well with the data obtained (Table 3), that ΔH < 0, ΔS > 0 with ΔG < 0, indicating an exothermically driven ion-exchange process. Ultimately, Pb^2+^ adsorption is favoured by low temperatures (Table 4).

**Table 3:** Adsorbent thermodynamic property data

Adsorbent	Enthalpy, ΔH (kJ mol^−1^)	Entropy, ΔS (J mol K^−1^)	*R*^2^
Alginate	− 118	395.65	0.97
Alginate-AA	− 77.4	272.27	0.99
Alginate-GO	− 85.2	295.23	0.99
Alginate-AA-GO	− 49.8	172.39	0.91
Amberlite	− 178	612.61	0.96

**Table 4:** Pb^2+^ adsorption characteristics for various adsorbents

Source	Adsorption capacity, q_max_ (mg g_−_^1^)	Reference
Extracted alginate adsorbents from seaweed sources		
*Sargassum natans*	213	This study
*Sargassum filipendula*	188	(Kleinubing et al. 2011)
*Laminara digitita*	372	(Papageorgiou et al. 2006)
*Laminara digitita*	218	(Wang et al. 2016)
*Laminara digitita*	109	(Varaprasad et al. 2020)
*Cystoseira barbata*	297	(Trica et al. 2019)
*Turbinaria ornata*	275	(Aden et al. 2019)
Sigma Aldrich (commercial)	250	(Shang et al. 2015)
Polysaccharide bio-based adsorbents		
Alginate/clay-g-poly (acrylic acid)	241–532	(Shahbazi et al. 2020)
Alginate/cellulose	89.5	(Wu et al. 2021)
Alginate/cellulose nanofiber	319	(Zhao et al. 2021)
Chitosan/nanocellulose	335	(Xu et al. 2021)
Tragacanth gum/graphene oxide	143	(Sahraei and Ghaemy 2017)
Glucan/chitosan	395	(Jiang et al. 2019)
Cellulose/acrylamide/acrylic acid	393	(Zhao et al. 2019)

For the composite beads, the enthalpy was 28–58% lower compared to the Alginate system. The decrease was attributed to the reduced amount of binding sites on the alginate matrix, mainly due to inhibition caused by the additives. This is particularly important for AA-GO, where more interactions between additives occur compared to all other absorbents, reducing the enthalpy by 58%. Amongst all absorbents, the Alginate system has the highest adsorption capacity of 213 mg g^−1^ which corresponds to the lowest enthalpy of − 117.9 kJ mol^−1^. Conversely, the alginate adsorbent blended with GO and AA exhibits a higher enthalpy of − 49.8 kJ mol^−1^ but lower adsorption capacity (179 mg g^−1^). Amberlite has the highest exothermic enthalpy but lowest adsorption capacity, attributed to the reactivity of the ${SO}_{3}^{-}$ group on the resin surface, compared to alginate systems where ion-exchange is facilitated via the “egg-box” model and coordinated within the alginate matrix. This observation has also been reported in literature (Alguacil 2002).

In literature, there are limited studies using beads fabricated from *Sargassum* for heavy metal ion remediation, which emphasizes the novelty of this research as a means to valorise *Sargassum natans*, which is required also from an environmental point of view, creating useful and valuable products. The performance of the Alginate system (213 mg g^−1^) fits the range (109–372 mg g^−1^) of adsorbents fabricated from commercial alginate sources (*Laminara digitata*), and performs better than that off *Sargassum filipendula* (188 mg g^−1^). However, compared to the alginate-polysaccharide-based blends, it shows slightly lower q_e_, which is attributed to differences in polymeric properties (β-d-mannuronic acid (M) residue: 1–4 linked α-l-guluronic acid (G) residue ratio, molecular weights, polymer conformity and chain length) and lower yield and quality of alginate extracted from *S. natans* compared to those from commercial sources.

Thus, future work is needed aiming to improve composite alginate through optimized blends, enhancing adsorption for instance through compatible additives such as other natural polysaccharides/blends (cellulose, carrageenan, chitosan, pectin, glucans). Such polymer blends provide additional binding sites and functional groups to improve the adsorption capacity of bio-based resins (Zhang and Tian 2020). This can be carried out via an optimization framework starting with a rigorous screening process followed by the use of composite technology and design of experiments (DOE) (Mohammed et al. 2023a), allowing for a systematic manipulation of additive composition as a function of alginate properties, facilitating controlled analysis and comparison of the resin performance under different conditions. This will ultimately illustrate how different additives can be complemented with alginates, to inform on heavy metal wastewater remediation.

### Packed bed application

**Fig. 6:**
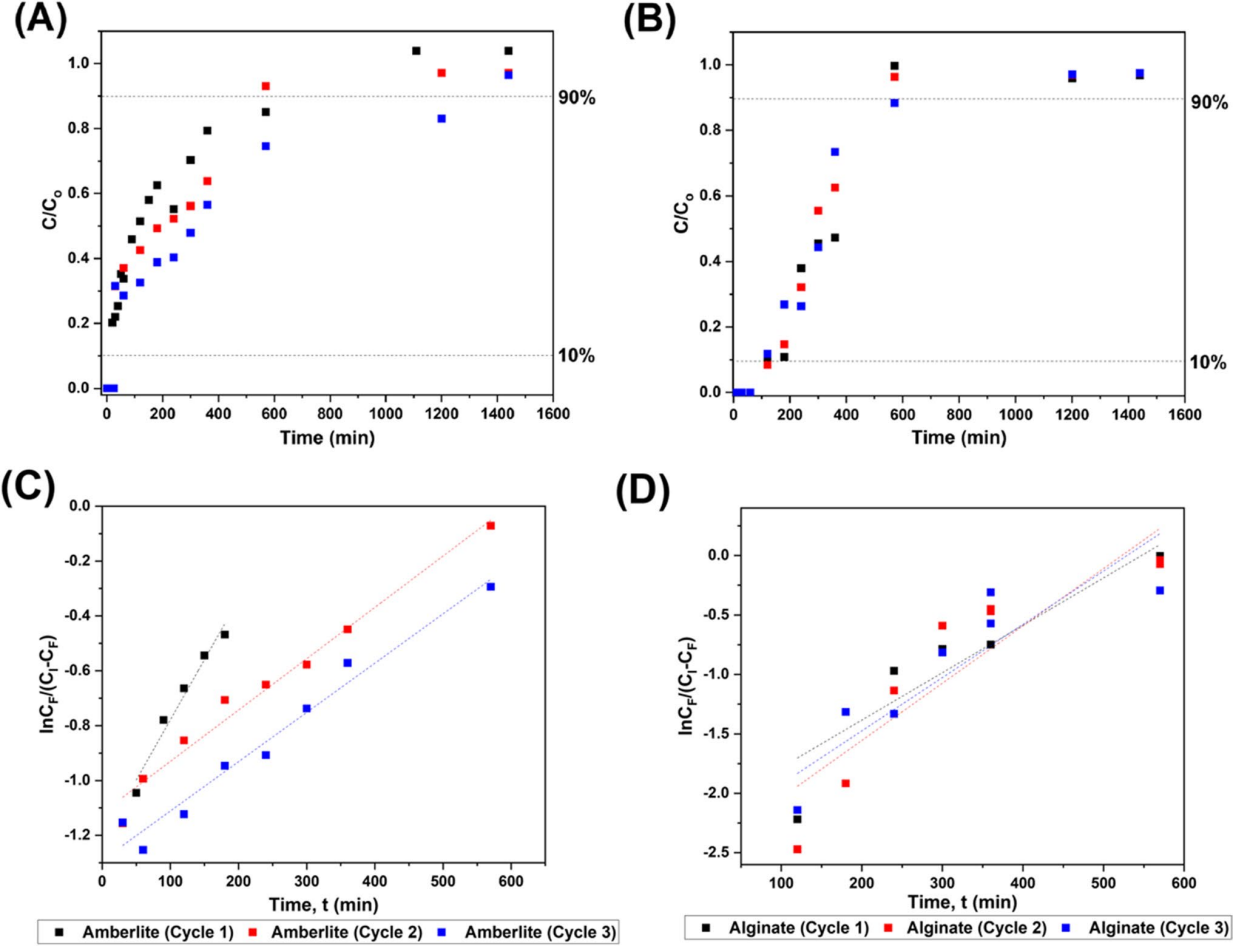
Performance curves represented over 3 operational cycles packed with **A** Amberlite, **B** Alginate, and linearized using the Yoon–Nelson correlations for **C** Amberlite and **D** Alginate respectively

While Alginate beads were proven successful at batch scale, a continuous process must be considered to validate its commercial applicability. Thus, pilot scale column experiments were evaluated following the schematic shown in Fig. 1 to determine the operational range for Pb removal by the Alginate system compared to its commercial counterpart (Amberlite). Figure 6 illustrates sigmoidal performance curves for (A) Amberlite and (B) Alginate as a function of rate of Pb adsorption through the bed. The bed becomes inoperable when the effluent stream concentration (i.e. rise exhaustion, C_F_) reaches 90–95% of the inlet concentration (C_I_) (Yahya and Odigure 2015), while break-through was determined at 10% C_I_. It was found that the Alginate system exhibits a longer operational time compared to Amberlite, which is an indication of the longevity of the resin. This correlates well with results of the batch studies, in which the Alginate system outperforms Amberlite as well.

Table 5 summarizes the performance of both Amberlite and Alginate systems during column experiments based on the linearized Yoon-Nelson models (*R*^2^ > 0.9 for all performance curves). To achieve a bed height of 3 cm, Alginate beads required a mass of approximately 15 times less than Amberlite. Despite this higher loading, the break-through times for Amberlite were 18, 24 and 29%, respectively, lower than that of Alginate for 3 consecutive cycles, respectively. The adsorption rates for Amberlite were higher than those of Alginate but resulted in lower removal efficiencies. This can be explained by examining the intrinsic properties of Amberlite. Rapid migration of ions to the surface of Amberlite was mainly driven by its high density and surface area (13.5 m^2^ g^−1^) compared to that of Alginate (0.4 m^2^ g^−1^) (Aghakhani et al. 2012). Higher density and specific surface area result in better binding and increased adsorption. After each cycle, the adsorbent was regenerated to remove adsorbed ions leading to mass loss at the surface. This mass loss decreases the overall loading of the column, resulting in a reduction of available binding sites (Patel 2020). This loss in performance is illustrated in Fig. 6 A and B, by the shortening of the break-through time after each cycle and faster bed saturation. Opposed to Amberlite, Alginate performance curves overlap (with minor changes in breakthrough time, illustrating a smaller loss in performance and resin mass (Table 5). Focusing on the results for adsorbent capacity between batch and column operations, column experiments performed marginally better giving a total adsorbent capacity of 248.64 mg/g–14% higher than batch experiments. On comparing the capacity of each adsorbent system to the effectiveness of the packed bed operation (Table 6), it is clear that the alginate system outperforms with a 3.5-fold higher adsorption capacity, 18% higher adsorption efficiency across the bed, 30% lower mass transfer zone and 11-fold longer operational time. Furthermore, these results show general consistency across regeneration cycles, with little change in performance across the packed bed. While further work is needed to optimize the operability of the packed be system, these results illustrate the impact of utilizing alginate adsorbents directly within industrial heavy metal ion wastewater systems.

**Table 5:** Packed bed adsorption performance characteristics for Amberlite and Alginate systems

Cycle	*R*^2^	Mass, m (g)	Yoon–Nelson proportionality constant, k × 10^−3^ (min^−1^)	Breakthrough, $\tau$(min)	q_e_ (mmol g^−1^)
Amberlite					
1	0.94	7.52	5.18	501	0.214
2	0.94	6.45	6.46	466	0.331
3	0.96	5.82	5.16	490	0.359
Alginate					
1	0.90	3.88	2.26	611	0.928
2	0.92	3.43	1.75	610	1.03
3	0.92	3.21	1.95	689	1.20

**Table 6:** Packed bed adsorption efficiency for Amberlite and Alginate systems

Cycle	q_B_ (mg g^−1^)	q_E_ (mg g ^−1^)	REM_b_ (%)	REM_e_ (%)	H_MTZ_ (cm)	t_B_ (h)	t_E_ (h)
Amberlite							
1	0.80	47.65	99.92	28.99	2.95	0.084	17.17
2	1.29	41.58	99.98	29.01	2.91	0.162	18.17
3	1.61	89.96	81.24	34.96	2.95	0.168	21.67
Alginate							
1	35.72	105.19	98.21	44.86	1.98	2.04	13.16
2	55.47	143.24	98.51	46.23	1.83	2.11	11.65
3	37.90	121.64	98.19	41.30	2.07	1.80	13.68

### Cycle time and scale-up of the adsorption process

The dimensions of the column and mass were estimated based on the treatment of wastewater at a flowrate (Q) of 1 m^3^ min^−1^ and Pb^2+^ concentration of 150 ppm. These calculations were based on a linear scale up of column experiments, adapted from García-Sánchez et al. (2016). Literature and respective model equations can be found in the Supplementary Information. Table 7 outlines the specific column properties for both systems.

**Table 7:** Comparison of the column properties and performance of a packed bed with alginate beads vs amberlite resin for Pb^2+^ removal

Column property	Alginate system	Amberlite
Design flowrate, Q (m^3^ min^−1^)	1	1
Bulk density, $\varphi$ (g cm^−3^)	1.13	3.97
Breakthrough time (min)	610	500
Height of column (cm)	3	3
Diameter of column, d (cm)	183	378
Mass of adsorbent, M (kg)	11.1	167.1

Our calculations showed that the mass of Amberlite required was 15 times higher than that of Alginate, based on the diameter required for the column of Amberlite systems being 2.1 times larger than that of the Alginate system, together with an 18% shorter operational time.

Our aim in this preliminary research phase was to comprehensively grasp the performance of alginate systems in comparison to existing commercial alternatives. Based on the performance of these column experiments as well as the scale-up models, it can be deduced that the Alginate resin system outperforms traditional packed bed applications for the removal of Pb^2+^ ions. Through this initial assessment, the alginate adsorbent system can be further refined and improved allowing for advanced packed bed modelling and mass transfer characteristics to be developed and tested in actual wastewater to further prioritize the use of these systems in industrial applications. This research also provides support for the suitability of Alginate resins, derived from *S. natans*, as an appropriate and efficient bio-based alternative for the remediation of Pb^2+^ contaminated wastewater. Economically, it serves as avenue to value creation and GDP growth across the Caribbean and the global bio-based adsorbent industry—directly competing with commercial alternatives. In terms of translational value, our experimental methodology and valorisation of a waste biomass provides technical knowledge aligned to bio-based heavy metal remediation, scale-up and productivity, which can be transferred and incorporated into the development of novel industries.

## Conclusions

Our work illustrates the potential use of *Sargassum* derived calcium alginate adsorbents for Pb^2+^ adsorption. Our results indicate an adsorption capacity of 213 mg g^−1^ at 20 °C and pH 3.5, surpassing conventional synthetic resins and rivals other seaweed-derived adsorbents. However, on the introduction of additives such as acrylamide and graphene oxide in the preparation of composite alginate adsorbents, while improving bead morphology, concurrently induced a 21–40% reduction in adsorbent efficiency compared to pure systems. Specifically, focusing on adsorbent capacity, column experiments demonstrated marginally superior performance, yielding a total adsorbent capacity of 248.64 mg/g – a 14% increase compared to batch experiments. Comparatively, the alginate system outperformed other adsorbent systems in packed bed operations, exhibiting a 3.5-fold higher adsorption capacity, 18% higher adsorption efficiency across the bed, 30% lower mass transfer zone and 11-fold longer operational time. Although the composite alginate systems investigated show inferior properties, compatible natural polysaccharide additives such as cellulose, carrageenan, chitosan, pectin and glucans emerge as a promising strategy for enhancing adsorption efficiency. In summary, these findings underscore the transformative potential of harnessing *Sargassum* for the production of bio-based materials, advocating for a paradigm shift towards environmentally conscientious resource utilization.

### Supplementary Information

Below is the link to the electronic supplementary material.

Supplementary file1 (DOCX 26 KB)

## Data Availability

Data will be made available upon request.
