# Supplementary material for: On the performance of Sargassum-derived calcium alginate ion exchange resins for Pb2+ adsorption: batch and packed bed applications
Source: Environ Sci Pollut Res Int. 2024 Apr 17;31(21):31224–39. doi: 10.1007/s11356-024-33314-w (PMC11096254; doi:10.1007/s11356-024-33314-w)
Supplement: Supplementary file 1 — Supplementary file1 (DOCX 26 KB) [file 11356_2024_33314_MOESM1_ESM.docx]

**On the Performance of *Sargassum* derived Calcium Alginate Ion Exchange Resins for Pb^2+^ Adsorption: Batch and Packed Bed Applications**

Akeem Mohammed^a^, Chantal Mohammed^a^ Andreas Mautner^b,c^, Matika Kistow^a^, Pooran Chaitram^a^, Alexander Bismarck^c^, Keeran Ward^d*^.

^a^Department of Chemical Engineering, The University of West Indies St. Augustine, Trinidad and Tobago

^b^Institute of Environmental Biotechnology, IFA-Tulln, University of Natural Resources and Life Sciences Vienna, Konrad-Lorenz-Str. 20, 3430 Tulln, 1180 Vienna, Austria

^c^Institute of Materials Chemistry and Research, Polymer and Composite Engineering (PaCE) Group, Faculty of Chemistry, University of Vienna, Währinger Straße 42, 1090 Vienna, Austria

^d^School of Chemical and Process Engineering (SCAPE), University of Leeds, Leeds, LS2 9JT, United Kingdom

*Corresponding author, Dr. Keeran Ward, School of Chemical and Process Engineering (SCAPE), University of Leeds, Leeds, LS2 9JT, United Kingdom

Email: [k.r.ward@leeds.ac.uk](mailto:k.r.ward@leeds.ac.uk)**;**

Tel: +44 7456 142816

**Supplementary Information**

**Methodology**

**S1: Rheology Study**

Sodium alginate concentration (2-8wt%) as well as GO (5-20% v/v) and AA (1-5 w/v%) were mixed and their viscosities measured at 25^o^C and a torque of less than 10% using a rheometer (Brookfield, AMTEK, USA. Beads were fabricated for each mixture following the procedure in the main article (**Section 2.3**). Each resin was imaged using a light microscope (Amscope). The maximum inscribed and circumscribed circles as well as the diameters were recorded. The sphericity was determined using equation [(1)](#equation14), as shown below

Sphericity, φ=Di/Dc (1)

Where *D*_i_ is the largest inscribed circle, and *D*_c_ is the smallest circumscribed circle.

**S2: Cycle time and scale-up**

The following equations were adapted from literature to determine the mass of adsorbent required, M (g) and diameter (cm) of the bed required. This scale-up requires knowledge of the breakthrough curve from a test column, which was operated at a predefined flow rate of Q_b_ (cm^3^ min^-1^) resulting in a breakthrough time of T_b_ (min). The bed volume was estimated using equation [(2)](#equation14).

Bed volume (BV) = $\frac{Q}{Q_{b}}$ (2)

The mass of the adsorbent, M (g) with a bulk density, $\varphi,$(g cm^-3^) for the design column was determined from equation [(3)](#equation11).

M = $\varphi\times BV$ (3)

The pilot scale breakthrough volume which occurs during a breakthrough time of $\tau$(mins)

(Yoon and Nelson model) was calculated using equation [(4)](#equation12).

V_B_ = Q_b_ x $\tau$ (4)

The volume of liquid treated per unit mass of adsorbent, $\tilde{V_{B}}$, was then determined using equation [(5)](#equation13).

$\tilde{V_{B}}= \frac{V_{B}}{M}$ (5)

The mass of adsorbent exhausted per hour, Mt, (g), and the breakthrough time, T (mins), for the design column was estimated using equation [(6)](#equation14) and [(7)](#equation15) respectively:

$M_{t}$ = $\frac{Q}{V_{B}}$ (6)

$T=\frac{M}{M_{t}}$ (7)

The volume, area, and diameter of the bed, V_B_ (cm^3^), for the design column was then estimated using equations [(8)](#equation16)-[(10)](#equation18):

V_B_ = Q_b_ x $T$ (8)

A = $\frac{V_{B}}{h}$ (9)

d = $\surd\frac{4\times A}{\pi}$ (10)

**Results and discussion**

Table S1: Mean parameter data for the Freundlich adsorption isotherm for Pb^2+^ onto pure alginate, alginate composites and commercial resin.

| Model Parameter | **Maximum Adsorption capacity, q_max_ (mg g^−1^)** | | | **Freundlich Constant, K_F_**  **(mmol g^−1^.(L mmol^−1^**$\boldsymbol{)}^{\frac{\boldsymbol{1}}{\boldsymbol{n}}}$ | | | **R^2^** | | |
| --- | --- | --- | --- | --- | --- | --- | --- | --- | --- |
| Temperature (°C) | 20 | 30 | 40 | 20 | 30 | 40 | 20 | 30 | 40 |
| Alginate | 212.8 $\pm6.4$ | 169.5 $\pm5.1$ | 101.0 $\pm3.0$ | 77.7 $\pm2.3$ | 95.0 $\pm2.8$ | 143.2 $\pm4.3$ | 0.74 | 0.74 | 0.87 |
| Alginate  +AA | 192.3 $\pm5.8$ | 123.5 $\pm3.7$ | 105.26 $\pm3.2$ | 81.04 $\pm2.4$ | 102.2 $\pm3.1$ | 158.1 $\pm4.7$ | 0.94 | 0.94 | 0.86 |
| Alginate  +GO | 172.4 $\pm5.2$ | 156.3 $\pm4.7$ | 119.1 $\pm3.5$ | 111.4 $\pm3.3$ | 103.3 $\pm3.1$ | 169.4 $\pm5.1$ | 0.90 | 0.94 | 0.86 |
| Alginate  +AA + GO | 178.6 $\pm5.4$ | 163.9 $\pm4.9$ | 116.3$\pm3.5$ | 93.67 $\pm2.8$ | 89.7 $\pm2.7$ | 130.3 $\pm3.9$ | 0.86 | 0.95 | 0.87 |
| Amberlite | 78.1 $\pm2.3$ | 61.4 $\pm1.8$ | 25.0 $\pm0.8$ | 21.7 $\pm0.6$ | 36.7 $\pm1.1$ | 41.5 $\pm1.2$ | 0.30 | 0.90 | 0.88 |

The linear regression (R^2^) for the Freundlich isotherm ranged from 0.3 - 0.95 over the temperature range and was thus, deemed not fitting to compare the performance of all absorbents. As a result, the Langmuir isotherm was used to fit the data which gave an R^2^ in the range of 0.92 – 0.99 and used for further comparison.
